# A Scan of Pleiotropic Immune Mediated Disease Genes Identifies Novel Determinants of Baseline FVIII Inhibitor Status in Hemophilia-A

**DOI:** 10.21203/rs.3.rs-3371095/v1

**Published:** 2023-10-18

**Authors:** Tom Howard, Marcio Almieda, Vincent Diego, Kevin Viel, Bernadette Luu, Karin Haack, Rajalingam Raja, Afshin Ameri, Meera Chitlur, Natalia Rydz, David Lillicrap, Raymond Watts, Craig Kessler, Christopher Ramsey, Long Dinh, Benjamin Kim, Jerry Powell, Juan Peralta, Ruayda Bouls, Shirley Abraham, Yu-Min Shen, Carlos Murillo, Henry Mead, Paul Lehmann, Eli Fine, Miguel Escobar, Satish Kumar, Sarah Williams-Blangero, Carol Kasper, Laura Almasy, Shelley Cole, John Blangero, Barbara Konkle

**Affiliations:** University of Texas Rio Grande Valley School of Medicine; University of California, San Francisco

**Keywords:** Hemophilia A, Factor VIII, Factor VIII gene (F8) mutations, Factor VIII inhibitors, Race/ethnicity, Candidate gene, Gene-centric association scan, Immune-mediated diseases, Immune-mediated disease genes and gene variants, Pleiotropic, Autoimmune-/autoinflammatory-disorders

## Abstract

Hemophilia-A (HA) is caused by heterogeneous loss-of-function factor (F)VIII gene (*F8*)-mutations and deficiencies in plasma-FVIII-activity that impair intrinsic-pathway-mediated coagulation-amplification. The standard-of-care for severe-HA-patients is regular infusions of therapeutic-FVIII-proteins (tFVIIIs) but ~30% develop neutralizing-tFVIII-antibodies called “FVIII-inhibitors (FEIs)” and become refractory. We used the PATH study and ImmunoChip to scan immune-mediated-disease (IMD)-genes for novel and/or replicated genomic-sequence-variations associated with baseline-FEI-status while accounting for non-independence of data due to genetic-relatedness and *F8*-mutational-heterogeneity. The baseline-FEI-status of 450 North American PATH subjects—206 with black-African-ancestry and 244 with white-European-ancestry—was the dependent variable. The *F8*-mutation-data and a genetic-relatedness matrix were incorporated into a binary linear-mixed model of genetic association with baseline-FEI-status. We adopted a gene-centric-association-strategy to scan, as candidates, pleiotropic-IMD-genes implicated in the development of either ³2 autoimmune-/autoinflammatory-disorders (AADs) or ³1 AAD and FEIs. Baseline-FEI-status was significantly associated with SNPs assigned to *NOS2A* (rs117382854; p=3.2E-6) and *B3GNT2* (rs10176009; p=5.1E-6), which have functions in anti-microbial-/-tumoral-immunity. Among IMD-genes implicated in FEI-risk previously, we identified strong associations with *CTLA4* assigned SNPs (p=2.2E-5). The *F8*-mutation-effect underlies ~15% of the total heritability for baseline-FEI-status. Additive genetic heritability and SNPs in IMD-genes account for >50% of the patient-specific variability in baseline-FEI-status. Race is a significant determinant independent of *F8*‐mutation-effects and non-*F8*-genetics.

## INTRODUCTION

Hemophilia-A (HA) is caused by X-linked factorVIII (FVIII) gene (*F8*) mutations and variable deficiencies in plasma FVIII coagulant activity (FVIII:C). Bleeding propensities in HA patients are inversely correlated with their baseline (i.e., untreated) FVIII:C levels and are classified as mild (5%£FVIII:C<40%), moderate (1%£FVIII:C<5%), or severe (FVIII:C<1%).^1,2^ Because recurrent hemarthrosis causes crippling joint disease, prophylactic infusions with plasma-derived (pd)-or recombinant (r)-therapeutic-FVIII-proteins (tFVIIIs)—called “products” herein—begin for severe HA patients when toddlers. However, ~30% of severe HA patients develop neutralizing anti-tFVIII-antibodies called “FVIII-inhibitors (FEIs)” which leave them refractory to these products and significantly increase morbidity and mortality.^3,4^

The immunogenicity of tFVIIIs is a complex trait with both environmental and genetic determinants deriving from several variable immunologically relevant characteristics of different products, treatment regimens, and patients.^5,6,7,8,9,10^ The highly heterogeneous causative-*F8*-mutations identified in unrelated HA patients has repeatedly been shown to comprise a potent genetic determinant of FEI risk.^5,6^ In a systematic review of data from >5,000 severe HA patients, Gouw *et al*. conducted a meta-analysis to estimate the relative risk of FEI development conferred by different categories of *F8* mutation types.^11^ In that report, the group of patients with any large deletion involving ³2 exons had an ~3–4x greater risk of FEI development than those with either distal or proximal intron (I) 22-inversions (invs), which account for ~45% of all newborns with severe HA, while the patients with I22-invs had an ~3x greater risk of FEIs than the patients with any missense single-base-substitution-mutation (SBSM), which is consistent with a contemporaneous report.^12^ Another recent study—conducted on a group of >1,200 non-severe HA patients with *F8* missense-SBSMs causing either moderate HA (~15%) or mild HA (~85%)—found comparable heterogeneity in FEI risk among subjects predicted to express endogenously almost 200 distinct mutant-FVIII-proteins, with most likely differing at only single amino acid residues.^13^ Taken together, these findings highlight the substantial *F8* mutation heterogeneity in relation to FEI risk, which we account for—for the first time—in this investigation.

An issue that needs to be addressed in genetic analysis of FEI risk is the widely observed fact that study samples tend to be composed of a sizeable fraction of related individuals. In certain studies, like the Malmö International Brother Study, a large fraction of genetic relatedness arises literally by design.^14^ In other studies, such as the current study, a lower level of genetic relatedness may arise in the sample because close relatives (e.g., brothers, male cousins, uncle-nephew pairs, and grandfather-grandson pairs) and even more distant relatives often receive medical care at the same specialized regional hemophilia treatment center (HTC). For example, in an investigation by Viel *et al*., for which HA patients were recruited from five HTCs in the southeastern US, 31% of the subjects were closely related to at least one other subject as recorded in their study questionnaires by the enrolling nurses.^15^ Thus, HTC-based recruitment appears more likely to be enriched for relatives as compared to random sampling of a population; importantly, this leads to the statistical problem of non-independence due to genetic relatedness.

The statistical methods we developed, and advocate herein, control for both *F8* mutation heterogeneity and genetic non-independence and are a straightforward extension of the linear mixed model analytic approaches that our group has been developing to study the genetics of complex diseases/traits.^16,17,18,19^ Specifically, we applied these methods to data from the Personalized Alternative Therapies for Hemophilia (PATH) study, which was designed to replicate and mechanistically clarify non-HA variants in *F8* found previously to be associated with the greater frequency of FEIs observed for HA patients self-reporting black-African (BA)-compared to white-European (WE)-racial/ethnic-ancestry.^15^ The results from several association studies of different candidate genes for autoimmune-/autoinflammatory-disorders (AADs)—referred to as immune-mediated diseases (IMDs) as they are caused by disorders of the adaptive and innate components of the immune system, respectively—have together identified variants in 25 such loci, which may contribute to the variable frequency of pathologic immunogenicity observed for the tFVIIIs infused in different HA patients.^20,21,22,23,24,25,26,27,28,29,30,31^ These results were often conflicting, however, and none have undergone true independent confirmation with functional validation. We used PATH genotyping data—from the single-nucleotide-polymorphisms (SNPs) and -variants (SNVs) assigned to the candidate ImmunoChip (IC) genes that have previously been implicated in the susceptibility to developing FEIs and/or AADs—in a gene-centric association-scan (GCAS) to identify novel and/or replicated IMD-genes that influence baseline-FEI-status.^32,33^

## MATERIAL AND METHODS

### Patients, questionnaires, and blood samples

Between January 2010 and December 2011, we invited North American HA patients managed clinically at any of 20 participating regional HTCs (14 in the US and six in Canada) to enroll in the IRB-approved PATH study based at the Los Angeles Veterans Affairs Medical Center (CA, USA) (IRB: 2009–091280) and Bloodworks Northwest (WA, USA) (IRB: 13018).^34^ For details on the PATH study’s cohort, inclusion and exclusion criteria, data collection instrument, blood samples obtained, and assays performed (see Supplemental Appendix).

### Bethesda assays for detecting and quantifying FEIs, determining historical- and baseline-FEI-status, and assessing FEI risk

As described by Pandey et al.,^34^ we determined each PATH subject’s: 1) historical-FEI-status by considering the results from all Bethesda assays^35,36,37^ performed before enrollment—during regularly scheduled HTC visits—which were obtained via medical chart review and provided to us in their questionnaires; and 2) baseline-FEI-status based on results from the single Bethesda assay performed on study entry. See Supplemental Appendix for details on the Bethesda assays performed, including the cutoffs used to interpret the results with respect to the presence or absence of FEIs, and assessment of FEI risk.

### Identification of HA-causing *F8*-mutations and creation/use of the shared *F8*-mutation-matrix

As described previously Pandey *et al*.,^34^ the *F8* mutations in HA patients were identified with three distinct *F8*-specific assays that involved Sanger resequencing, RT-PCR, and PCR,^38^ which were also employed in the Pharmacogenetics of Inhibitor Risk study by Viel *et al*., except that a long-range PCR (instead of an RT-PCR) assay was employed to detect I22-invs.^15^ See Supplemental Appendix for details on the three assays performed, including the specific *F8* mutation type(s) identified (or not identified) by each and their QC criteria, as well as the design/creation and use of the *F8*-mutation-matrix.

### Preparation and quality control of IC genotyping data

Genomic-DNA from each PATH subject was subjected to genotyping at the nearly 200,000 sequence variations that can be interrogated simultaneously on the IC (Illumina; San Diego, CA, USA).^32,33,39^ See Supplemental Appendix for details on the genotyping assay including the: IC version used; types and chromosomal locations of the variations genotyped; QC criteria for variations yielding high-quality genotypes; and the numbers of genotyped variations used in the (i) empiric-kinship/genetic-relationship matrix and (ii) GCAS of candidate pleiotropic-IMD-genes.^40^

### IC-based empiric-kinship/genetic-relationship matrix

All IC-variations with high-quality genotypes (n=137,776) were used to compute the pairwise genetic correlation between the PATH study subjects and to create the empiric kinship/genetic relationship matrix. See Supplemental Appendix for details on creating and utilizing the empiric kinship/genetic relationship matrix.

### Novel gene-centric association-scan (GCAS) of pleiotropic-IMD-genes

Because results from prior studies of immune-response-genes/-gene-variations have been conflicting and none have been confirmed with functional validation, we employed new statistical methods—that, as described under the next sub-header, controlled for *F8* mutation heterogeneity and genetic non-independence—in a GCAS focused on pleiotropic-IMD-genes, which met the extra criterion for candidates previously implicated in the development of FEIs and/or AADs.^20,21,22,23,24,25,26,27,28,29,30,31,32,33,^ See Supplemental Appendix for more details.

### Linear-mixed model with an additional *F8*-mutation-type component to evaluate risk of being FEI+ on study entry

We analyzed baseline-FEI-status (Yes versus No) for associations with the subsets of candidate IC-sequence-variations under a binary linear mixed model^16,17^ given as follows:

$$y_{i}=a+b\prime x_{i},$$

where for the -th individual denotes the baseline-FEI-risk value, denotes the vector of covariates, is the intercept parameter, and is the vector of regression coefficients. See Supplemental Appendix for more details on how we: 1) selected/examined covariates; 2) accounted for potential sources of non-independence; 3) derived odds ratios (ORs); 4) calculated/used heritabilities; and 5) analyzed the genetic effect of each high-quality SNP and SNV—comprising the subsets of variations which were assigned by consensus of the IC-consortium to one of the candidate pleiotropic-IMD-genes known or suspected to influence the development of ³2 distinct AADs (n=125) and/or FEIs (n=22)—for its contribution to baseline-FEI-status by means of a likelihood-ratio-test as implemented in SOLAR.

## RESULTS

### Descriptive statistics of the PATH cohort

**Figure 1:**
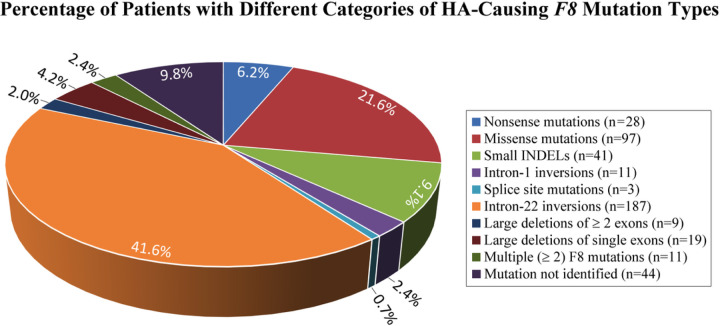
Pie chart showing the number of PATH study subjects that fall into each of the eight broadly distinct categories of HA-causing *F8*mutation types including: nonsense mutations, i.e. nonsense single-base-substitution mutations (SBSMs); missense mutations, i.e. missense-SBSMs; small insertions and/or deletions (INDELs); intron (I) 1-inversions (invs); consensus splice site mutations, i.e. splice site SBSMs; I22-invs; large deletions involving multiple exons (³ 2 exons); and large deletions of single exons or smaller (£ 1 exon). It also charts the number of HA patients that had ³ 2 distinct causative-*F8*-mutations and the number of patients whose *F8*mutations were not identified. Note that the three assays employed herein are not able to identify the specific causative-*F8*-abnormality in HA patients with mutations in either of the following three categories: (i) large duplications of one or more exons; (ii) deep intronic SBSMs; and (iii) deep intronic small INDELs.

We conducted the PATH study on a cohort of 450 HA patients who self-reported either BA (45.8%) or WE (54.2%) racial/ethnic ancestry and were from the US or Canada (Table 1). The proportion of BA and WE subjects who had ever developed a FEI of any titer (AT), ${\text{FEI}}_{{\text{AT}}^{\text{+}}}$, prior to study entry (i.e., had a positive historical-FEI-status) was 35.1% and 26.6%, respectively (Table 1a), while the proportion of BA and WE subjects who had AT FEI (${\text{FEI}}_{{\text{AT}}^{\text{+}}}$) at study entry (i.e., had a positive baseline-FEI-status) was 21.5% and 15.9%, respectively (Table 1b). Using Fisher’s method for combining p-values for the chi-square tests (p=0.06 and p=0.14, respectively), the joint tabular data are marginally not significant (p=0.06) but trend in the expected direction of greater prevalence of FEIs in HA patients with BA-racial/ethnic-ancestry.^15^ We identified causative *F8* mutations in 406 subjects (90.2%): 188 of the 206 BA (91.3%) and 218 of the 244 WE (89.3%) subjects (Figure 1). The two most common mutations were I22-invs^11,15,34,41,42,43^—identified in 187 subjects (41.6%) including 70 (34.0%) and 117 (48.0%), respectively, with BA- and WE-racial/ethnic-ancestry—and the missense-SBSMs,^5,10.11,12,14,44^ although the later are occasionally nonsynonymous (ns)‐SNPs, especially when found in the FVIII B-domain.^8,15,34,43,45^ We found *F8* missense-SBSMs in 97 subjects (21.6%) (Figure 1), including 60 (of 206) with BA (29.1%) and 37 (of 244) with WE (15.2%) racial/ethnic-ancestry. These 97 subjects carried 61 distinct *F8* ns-SBSMs, including 15 that were previously unknown when the PATH study was conducted: 79E>Q; 155T>S; 414N>D; 669T>P; 964V>I; 979H>Q; 1193K>N; 1460G>D; 1499H>Y; 1776R>K; 1801E>K; 1846D>V; 1966R>Q; 1969E>K; and 2143P>Q.

Because the baseline-FEI-status of HA patients has never been investigated genetically previously, we estimated its heritability for the first time herein. (Though the genetics associated with FEI risk has been reported on extensively previously, the dependent variable in prior studies has been “lifetime-FEI-status”, i.e., what we refer to here as historical-FEI-status, which is: ‘YES’, if a patient has ever developed FEIs, of AT, at any time in life, irrespective of whether or not a FEI is present at ‘baseline’, on study entry; or ‘NO’, if a patient has never developed FEIs, of AT, at any time in life, including at ‘baseline’, on study entry.) We found that the residual additive genetic heritability for the presence of FEIs of AT (${\text{FEI}}_{{\text{AT}}^{\text{+}}}$) on entry into the PATH study versus their absence (${\text{FEI}}_{{\text{AT}}^{-}}$) was 0.47 (p<0.05) and the *F8*-mutation-specific heritability was 0.08 (p<0.05), the latter of which comprised ~15% of the total observable heritability (0.08/0.08+0.47). We leveraged the substantial significant additive genetic and *F8*-mutation-specific heritabilities for baseline-FEI-status to perform a GCAS in which the 101 distinct pleiotropic-IMD-genes on the IC—that met the criterion of having previously been implicated in the risk of developing either two or more distinct AADs (n=76) or FEIs and at least one AAD (n=21), or both (n=4)—were screened as candidate determinants for the presence or absence of FEIs (on study entry) in these previously-treated-patients (PTPs) with HA.

### Novel and replicated IMD-genes/-gene-variants identified using the candidate gene/GCAS approach

**Figure 2:**
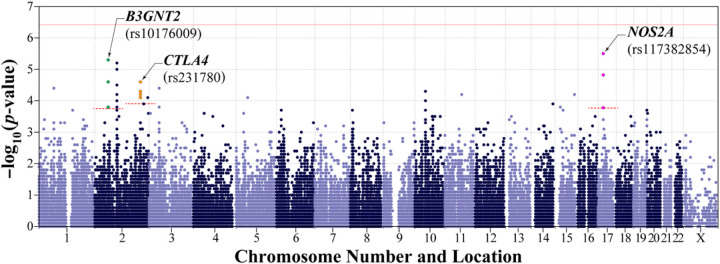
Manhattan plot of association results from an ImmunoChip (IC)-wide scan of baseline-FEI-status, highlighting the 10 SNPs assigned to the three pleiotropic-IMD-genes—including *B3GNT2* on Chr2 (three solid green circles), *CTLA4* on Chr2 (four solid orange circles), and *NOS2A* on Chr17 (three solid purple circles)—found to be significantly associated in the gene-centric scans shown at higher resolution in Figure 3. Each light- or dark-blue filled circle indicates one of the subset of 137,766 IC SNPs that yielded high-quality genotypes (i.e., passed QC as detailed in the Supplemental Appendix) but were not significantly associated with baseline-FEI-status at a significance threshold Bonferroni corrected for the multiple testing of the IC-wide set of 137,776 total SNPs that passed QC, i.e., they are all below the solid horizontal red line shown at the −log _10_ (*p*-value) of 6.44, which would be the corrected threshold accounting for all successfully genotyped ImmunoChip SNPs. (Note that Bonferroni correction is defined as the nominal significance threshold, i.e., a p-value < 0.05, divided by the total number of genotyped SNPs analyzed, which, in this case, was 137,776.) All 137,776 genotyped SNPs were used to create the empiric-kinship matrix that was utilized to account for the non-independence of data (due to both the known and unknown relationships among the study subjects) and estimate the heritability of baseline-FEI-status. The 10 SNPs significantly associated with baseline-FEI-status in the gene-centric scans included three, four, and three assigned respectively to *B3GNT2*, *CTLA4*, and *NOS2A* which fell above their respective −log _10_ (*p*-value) thresholds of 3.75, 3.86, and 3.76 that are represented by the three horizontal red dashed lines (see Figure 3 for more details).

**Figure 3:**
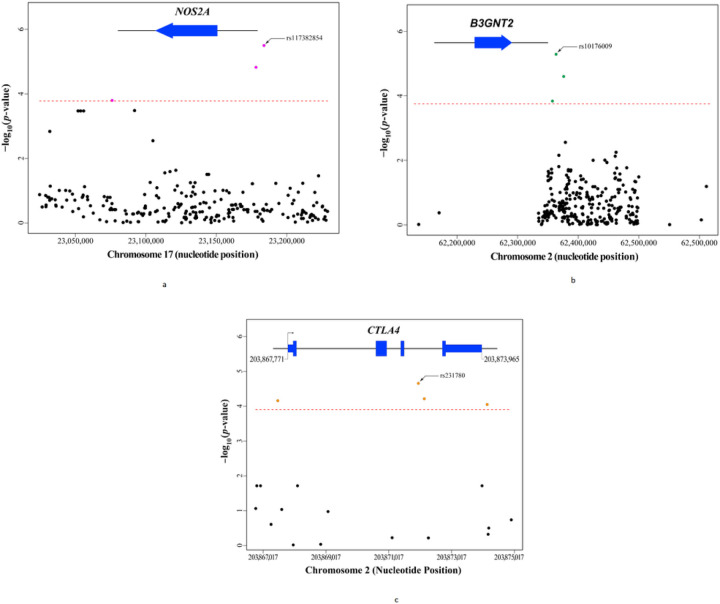
Focused Manhattan plots of the GCAS results from the three pleiotropic-IMD-genes with SNPs that demonstrated significant genotype-specific associations with baseline-FEI-status. (A) Chr17 long-arm location of *NOS2A*—and its centromerically-oriented transcription unit—spanning antisense-strand nucleotides 23,107,919–23,151,682, is illustrated with the solid blue arrow. The subset of 287 SNPs assigned to *NOS2A* that passed QC and reside within the Chr17 coordinates shown on the X-axis are all indicated with dark-blue circles except for the three purple circles which represent the SNPs associated with baseline-FEI-status in the GCAS as indicated by their position above the red dashed line designating the −log _10_ (*p*-value) threshold of 3.76. Two of these three SNPs—including rs117382854, the top association signal—are in the 5’-regulatory-region of *NOS2A*, while the third is in the 3’-flanking DNA, downstream of the sequence encoding the cleavage/polyadenylation site in its primary transcript. (B) Chr2 short-arm location of *B3GNT2*—and its centromerically-oriented transcription unit—spanning sense-strand nucleotides 62,196,114–62,224,730, is illustrated with the solid blue arrow. The subset of 281 SNPs assigned to *B3GNT2* that passed QC and reside within the Chr2 coordinates on the X-axis are all indicated with dark-blue circles except for the three green circles which represent the SNPs associated with baseline-FEI-status in the GCAS as indicated by their position above the red dashed line designating the −log _10_ (*p*-value) threshold of 3.75. All three of these SNPs (including rs10176009, the top signal) are in the 3’-flanking DNA, downstream of the sequence encoding the cleavage/polyadenylation site in its primary transcript. (C) Chr2 long-arm location of *CTLA4*—and its telomerically-oriented transcription unit—spanning sense-strand nucleotides 203,867,771–203,873,965, is illustrated with the solid blue arrow. The subset of 362 SNPs assigned to *CTLA4* that passed QC and reside within the Chr2 coordinates on the X-axis are all indicated with dark-blue circles except for the four orange circles which represent the SNPs associated with baseline-FEI-status in the GCAS as indicated by their position above the red dashed line designating the −log _10_ (*p*-value) threshold of 3.86. Two of these four SNPs (including rs231780, the top signal) are in I3 of *CTLA4*, while one is in its proximal promoter and one is in 3’-flanking DNA, downstream of the sequence encoding its primary transcript’s cleavage/polyadenylation site.

Using the IC-genotypes from PATH subjects, we found that baseline-FEI-status—i.e., the presence versus absence of FEIs at enrollment (${\text{FEI}}_{{\text{AT}}^{\text{+}}}$ vs ${\text{FEI}}_{{\text{AT}}^{-}}$)—was significantly associated with SNPs assigned to each of three pleiotropic-IMD-genes, *NOS2A*, *B3GNT2*, and *CTLA4* (Figure 2), with *NOS2A* variations yielding the strongest signals (Table 2). Located on the proximal long-arm of chromosome (Chr)17, *NOS2A* comprises a centromerically-oriented, 27-exon-containing transcription unit that spans antisense-strand nucleotides 23,107,919–23,151,682 (NCBI36/hg18) (Figure 3A) and encodes the inducible isoform of nitric oxide (NO)-synthase (NOS) 2, which produces NO, a labile uncharged lipophilic gas molecule that freely passes phospholipid bilayers. Because NO signals by activating soluble guanylyl cyclase, which in turn increases synthesis of cGMP from cellular GTP, NOS2 is a vital regulator of several physiologic processes especially those in the immune system related to host defense as NO has potent microbicidal- and tumoricidal-actions.^46,47^ Though *NOS2A* had not previously been implicated in the development of FEIs, nor in their persistence and/or resolution, it likely influences the development of at least five AADs, including multiple sclerosis (MS), insulin-dependent diabetes mellitus (IDDM), rheumatoid-arthritis (RA), systemic-lupus-erythematosus (SLE) and myasthenia gravis (MG).^46,47,48,49,50,51^ We identified *NOS2A*’s involvement in the immunogenicity of tFVIIIs via three SNPs (Figure 2)—with the top being rs117382854 (*p*=3.2E-6), a C>G substitution at nucleotide 23,183,786, ~34 kbp upstream of its translation initiation-codon (Figure 3A)—whose minor alleles (MAs) comparably increased risk of having FEIs of AT on study entry (Table 2).

Like *NOS2A*, *B3GNT2* likely influences the risk of at least five AADs—including IDDM, RA, Graves’-disease (GD), ankylosing spondylitis (AS), and Crohn’s disease (CD)^52,53,54,55,56^—and it has not previously been implicated in the development, persistence, and/or resolution of FEIs. Located on the short-arm of Chr2, *B3GNT2* comprises a centromerically-oriented, two-exon-containing transcription unit spanning sense-strand nucleotides 62,196,114–62,224,730 (GRCh38/hg38) (Figure 3B) and encodes the widely expressed UDP‐GlcNAc:bGal b-1,3-N-acetyl-glucosaminyltransferase-2 enzyme (B3GNT2), the major golgi-resident glycosyltransferase which catalyzes the addition of repeating-polylactosamine-chains to N-glycan core structures of transmembrane- and secreted-glycoproteins.^57^ It is known that excessive immune responses are suppressed by poly-*N*-acetyl-lactosamine and that deletion of *B3GNT2* yields hypersensitive/hyperresponsive immunocytes by markedly reducing its levels on cell-surfaces.^57^ *B3GNT2* was identified here via three *B3GNT2*-assigned SNPs (Figure 2)—with the top being rs10176009 (p=5.1E-6), a C>A substitution at nucleotide 62,282,826, ~59 kb downstream of its stop-codon (Figure 3B)—whose MAs comparably increased risk of having FEIs at baseline (Table 2).

Under the GCAS-approach, we found that the presence of FEIs on study entry (${\text{FEI}}_{{\text{AT}}^{\text{+}}}$) versus their absence (${\text{FEI}}_{{\text{AT}}^{-}}$) was strongly associated with four *CTLA4*-assigned SNPs (Figure 2), with rs231780 yielding the strongest signal (*p*=2.3E-5) (Table 2), and suggestively associated with rs7528265, a SNP assigned to the interleukin (IL)-10 gene (*IL10*) (*p*=3.7E-3), which is closely linked to the three other Chr1 genes of the *IL10*-superfamily, i.e., *IL19*, *IL20*, and *IL24* encoding IL-19, IL-20, and IL-24 (Table 2). Like *NOS2A* and *B3GNT2*, both *CTLA4* and *IL10* had previously been implicated in multiple AADs—including, e.g., RA, IDDM, and SLE^58,59,60,61,62,63^—but both had also been confirmed genetically to play a role in the development of FEIs.^20,22,23^ Located on the long-arm of Chr2, *CTLA4* comprises a telomerically-oriented, four-exon-containing transcription unit which spans sense-strand nucleotides 203,867,771–203,873,965 (GRCh38/hg38) (Figure 3C) and encodes the T-cell receptor—cytotoxic T-lymphocyte-associated-protein-4 (CTLA4)—that transduces inhibitory signals to down-regulate immune responses.^63^ SNP rs231780 (203,871,974 A>G)—which is located at nucleotide position +475 of *CTLA4* I3—revealed a statistically-significant, genotype-specific decrease in FEI risk (beta = −0.555) (Table 2). (Note that dbSNP lists “G” as the major allele of rs231780, but “A”—which was coded in the GCAS as the MA and yielded a positive beta, i.e., increased risk—is the major allele.) The other *CTLA4*-assigned SNPs associated with baseline-FEI-status also reside in functional gene regions including its proximal promoter, 3’-genomic-DNA, downstream of the cleavage-/polyadenylation-site encoding sequences, and I3 (Figure 3C). *IL10* is a centromerically-oriented, five-exon-containing transcription-unit on the long-arm of Chr1 that spans antisense-strand nucleotides 206,767,602–206,772,494 (GRCh38/hg38) (not shown). This pleiotropic-IMD-gene is expressed by regulatory T-cells and encodes IL-10, an anti-inflammatory cytokine that helps balance immune responses, allowing clearance of infectious microbes but minimizing host damage.^64^ The *IL10*-superfamily was identified via rs7528265 (206,845,818 C>T)—an *IL10*-assigned SNP located ~73kbp upstream from its initiation-codon—which revealed a suggestive genotype-specific increase in FEI risk (p=3.7E-3) (not shown).

#### Multivariate logistic regression odds ratios

**Figure 4:**
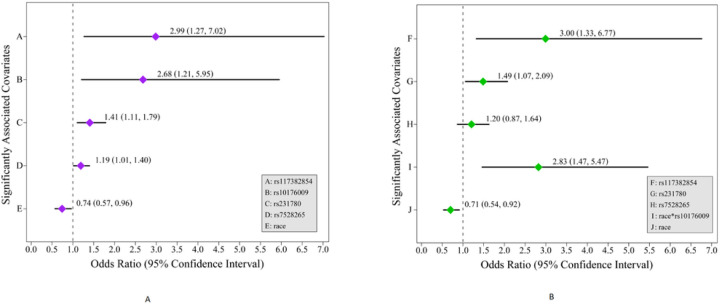
Plots of ORs of the effect of IMD-genes/-gene-variants and race as covariates on the outcome of baseline-FEI-status under the additive (A-E) and interaction (F-J) models. (A) Under the additive model, the ORs for covariates A-E are indicated with purple diamonds and their 95% CIs are indicated with black horizontal lines where, for each covariate, the 95% CI LB and UB are represented by the left and right ends of the line, respectively. The OR and 95% CI—reported as OR (95% CI LB, 95% CI UB)—are shown for (A) rs117382854, (B) rs10176009, (C) rs231780, (D) rs7528265, and (E) race. (B) Under the interaction model, the ORs for covariates F-J are indicated with green diamonds and their 95% CIs are indicated with black horizontal lines where, for each covariate, the 95% CI LB and UB are represented by the left and right ends of the line. The OR and 95% CI are shown for (A) rs117382854, (B) rs231780, (C) rs7528265, (D) race*rs10176009, and (E) race.

Having identified a novel role for *NOS2A* and *B3GNT2*¾and verified the previously implicated role for *CTLA4* and *IL10*¾in FEI risk, we incorporated the top SNP assigned to each of these pleiotropic-IMD-genes altogether in a model, along with race, to evaluate their joint ability to predict baseline-FEI-status where all effects are additive. The OR and 95%-confidence interval (CI) lower- and upper-bound (LB and UB)¾reported as OR (95% CI LB, 95% CI UB)¾for rs117382854, rs10176009, rs231780, rs7528265, and race, respectively, were 2.99 (1.27, 7.02), 2.68 (1.21, 5.95), 1.41 (1.11, 1.79), 1.19 (1.01, 1.40), and 0.74 (0.57, 0.96) (Figure 4A, covariates A-E), which indicate that the MAs of three of these four IMD-gene-variations (i.e., *NOS2*, *B3GNT2*, and *IL10*) increased the risk of having FEIs of AT on study entry while the true MA of *CTLA4* and WE-racial/ethnic-ancestry were protective.

Because the main goal of the PATH study was to confirm and further clarify the influence of race on FEI development, we sought to explicitly evaluate whether its role is mediated in part via genetic effects by Bayesian model selection of all possible interactions of race with these four pleiotropic-IMD-gene-variations.^65^ The best model identified involved a single interaction term between race and *B3GNT2* (i.e., rs10176009), where rs117382854, rs10176009, and race, by itself, still exerted significant additive effects (Figure 4B, covariates F-J). The ORs and 95% CIs for rs117382854, rs231780, rs7528265, race×rs10176009, and race alone are respectively 3.00 (1.33, 6.77), 1.49 (1.07, 2.09), 1.20 (0.87, 1.640), 2.83 (1.47, 5.47), and 0.71 (0.54, 0.92).

## DISCUSSION

Prior studies aimed at identifying inherited determinants of FEI development in PTPs have exclusively examined historical-FEI-status and¾except for a few which recently employed genome-wide scans^66,67,68^¾have used candidate-gene-based approaches that focused on sequence variation in or near either *F8* (i.e., mutation typesand/or non-HA-causing ns-SNPs)^5,6,7,8,9,10^ or essential immune system genes.^20,21,22,23,24,25,26,27,28,29,30,31^ While findings from these studies have clearly demonstrated the importance of *F8* mutation type, they have been conflicting with respect to the role played by *F8* ns-SNPs and IMD-genes/-gene-variants.^9,20,21,22,23,24,25,26,27,28,29,30, 31,34,44,69,70,71,72^ Specific mechanisms underlying the differential effects of some *F8* mutation types are being elucidated and appear to variably involve interrelated processes regulating immunologic-tolerance (IT) and -reactivity (IR).^8,11,13,34,43,44^ In this context, IT refers to whether a given patient’s abnormal *F8* encodes a polypeptide(s)¾if indeed one or more is encoded¾that, except for the residue(s) resulting from the specific mutation(s), contain the entire amino acid sequence of his tFVIII, i.e., the endogenous material needed to induce IT to his “self” FVIII, which theoretically would provide preexisting-immunologic-unresponsiveness to the identical portions of his tFVIII. The process of IR relates to whether one of the limited number of distinct allotypes in the repertoire of HLA-class-II (HLAII) molecules expressed by dendritic cells and B-cells in a given HA patient (i.e., which ranges from 3–12) is able to present—as a neoepitope to his CD4 T-cells, threshold levels of at least one “foreign” peptide liberated from his tFVIII via intracellular proteolytic processing.^73,74^ We presented findings from the first study of baseline-FEI-status, a novel outcome for PTPs that appears to be affected distinctly by what we have coined the “immunogenicity balance”—a complex balance of processes relating to IT and IR—than historical-FEI-status as the presence of FEIs on study entry likely also interrogates determinants influencing whether an immune response is persistent or resolves in addition to its induction/development.

### Accounting for *F8* mutational heterogeneity and genetic relatedness

We identified causative-*F8*-mutations in 406 of the 450 HA patients enrolled in PATH (90.2%), including 188 of the 206 with BA (91.3%) versus 218 of the 244 with WE (89.3%) racial/ethnic-ancestry. Although *F8* abnormalities were not found in 44 patients (9.8%)—for the various reasons described above, which were likely variably at play in prior studies as causative-*F8*-mutations are frequently not identified in between 7.5–15% of subjects—these were comparably disbursed across the groups self-reporting BA-versus WE-ancestry, i.e., 8.7% versus 10.7%, respectively. The *F8* abnormalities identified in PATH were highly heterogeneous—comprising 132 distinct loss-of-function mutations—across both groups of subjects. This included the highly-recurrent bi-allelic I22-invs and the allelically highly-diverse missense-SBSMs—the two most common causative-*F8*-mutation types—which, among the US subjects respectively accounted for 31.9% and 32.4% versus 42.7% and 19.0% of those with BA-versus WE-racial/ethnic-ancestry, respectively. This extreme heterogeneity among causative-*F8*-mutations has been found in all racial and ethnic groups of patients studied thus far.^15,67,68,75,76^ To account for mutational heterogeneity appropriately/accurately, we also quantified the number of causative-*F8*-abnormalities in each of the other categories identified. Because this heterogeneity has not been accounted for rigorously in prior studies of FEIs, we achieved this herein by employing a shared causal-*F8*-mutation matrix to estimate the mutational effect. Our linear mixed model can be conceptualized in terms of the: 1) signal-to-noise ratio, where statistical noise arises from both the (i) non-independence that derives from genetic relatedness in the samples, and (ii) large degree of causative-*F8*-mutational heterogeneity (and is accounted for by the random effects part of the model); and 2) main genetic signals of interest that result from the SNPs/SNVs (and is accounted for by the fixed effects part of the model).

In association studies with a non-negligible number of related patients (e.g., full- and half-sibs, first-cousins, and avuncular-relations), it is important to account for the genetic relatedness of the subjects. However, the extensive amount of genetic relatedness existing in many prior studies of FEIs has not been adequately accounted for. Two types of genetic relatedness exist from familial relationships among subjects including those known and those unknown but obtained from empirical estimates of relatedness over a panel of genetic markers. Our empirical kinship matrix estimates both types, as they belong to the same relatedness continuum (see Supplementary Figure 1). The extent of relatedness in such studies results in a tacit violation of the independence-of-data assumption. Therefore, modeling this non-independence is a crucial feature of our approach. It is important to emphasize that accounting for the non-independence due to both relatedness and causative-*F8*-mutation heterogeneity helps to optimize the efficiency of parameter estimation in the fixed effects.

### Fixed effects modeling of baseline-FEI-status

#### Novel IMD-genes/-gene-variants involved in FEI risk

Using our GCAS approach and the high-quality genotyped IC-SNPs/-SNVs assigned to the 101 candidate-genes interrogated, we discovered a novel role for *NOS2A* and *B3GNT2*—two pleiotropic-IMD-genes known to influence the development of ³2 AADs but not FEIs—as determinants of baseline-FEI-status. We also discovered that *CTLA4* and *IL10*—two additional pleiotropic-IMD-genes known to influence the development of (i) ³2 AADs, and, in contrast to *NOS2A* and *B3GNT2*, (ii) FEIs, i.e., historical-FEI-status—are determinants of baseline-FEI-status via respectively four SNPs in *CTLA4* and one novel SNP in the *IL10*-superfamily, despite the fact that both genes have previously been implicated and (now) independently confirmed to play a role in FEI risk. The novel SNP of *CTLA4*, which is in I3, remained significantly associated after correction for multiple testing (data not shown). The novel SNP of *IL10*, which is in the upstream regulatory sequence, was marginally associated with FEIs but became only suggestively associated after multiple testing correction (data not shown).

#### Role of race in FEI risk

Under the additive model, race had an independent effect on FEI risk. Specifically, we observed that HA patients self-reporting BA-racial-ancestry had a higher prevalence of FEIs (i) historically (35.1%) compared to the patients with WE-racial-ancestry (26.6%), and (ii) at study entry (21.5%) compared to the WE-racial-ancestry patients (15.9%), which is consistent with what is commonly reported (although marginally not significant: p=0.06).^10,14,15,71,72,77,78,79^ Of note, past studies that identified self-reported BA-racial-ancestry to be a determinant of risk for FEIs, utilized historical-FEI-status only, not baseline-FEI-status.

Under the interaction model, we identified an interaction between race and rs10176009 (i.e., 62,282,826 C>A), a *B3GNT2*-assigned SNP located in the flanking 3’-gDNA of this pleiotropic-IMD-gene, which is known to influence susceptibility to at least five distinct AADs. This interaction may be interpreted as suggesting that the MA of this SNP (‘A’) increases the risk of having any titer FEI (${\text{FEI}}_{{\text{AT}}^{\text{+}}}$) at baseline in HA patients but only in patients with BA-racial-ancestry as it is rare in those with WE-racial-ancestry.

#### Potential role of rare genetic variants in FEI risk

Because the IC was primarily constituted of IMD-gene-variations found in GWASs to influence susceptibility to ³1 AADs,^32,33^ and the genotyping-platforms used in these GWASs were comprised predominantly of by common (MA-frequencies [MAFs] >5%) and less common (1%£ MAFs £5%) SNPs, the majority of variations analyzed herein represent such SNPs (Supplementary Figure 2). Thus, rare SNVs (MAFs <1%) are infrequent on the IC. Moreover, as we have accounted for most of the *F8*-genetic-effects (i.e., mutation effect), the 47% additive genetic heritability may therefore be largely the result of rare variants. This is consistent with the important role that rare variants are now widely recognized as playing in complex traits.^80^ Notably, while MAs of the first five SNPs in Table 2—which were assigned to three pleiotropic-IMD-genes (*NOS2A*, *B3GNT2*, and *CTLA4*) and found in our GCAS to have the strongest associations with baseline-FEI-status—are of the less common or common types in patients with one racial-ancestry (i.e., two *NOS2A*-assigned SNPs in WE subjects; two *B3GNT2*-assigned SNPs and one *CTLA4*-assigned SNP in BA subjects), they are rare SNVs in subjects of the other racial-ancestry (i.e., two *NOS2A*-assigned SNVs in BA subjects; two *B3GNT2*-assigned SNVs and one *CTLA4*-assigned SNP in WE subjects).

### Limitations

A limitation of PATH is under representation by mild and moderate HA patients in the cohort. Typically, up to 40% of subjects have non-severe HA when enrollment of patients—in developed countries, such as, e.g., in the US and Canada, which participated herein—occurs (i) during their scheduled bi-annual HTC appointments, and (ii) cross-sectionally, independent of their baseline-HA-severity and historical-FEI-status.

As this is a study almost exclusively of PTPs, with most being older than 18 years of age, the tFVIII intravenous-infusion histories are invariably inaccurate with respect to important parameters such as the number of exposure days of a given tFVIII administered previously and the age at first infusion of the initial tFVIII. Moreover, since most of the patients (almost 90%) have severe HA, the vast majority will have been on multiple different tFVIII products, including both pdFVIII and rFVIII, and within both of these broad categories of tFVIIIs, they likely will have received different brands (i.e., for pdFVIII products both those with and without pdVWF, and for rFVIII products, both those that are native full-length and those that are non-native with engineered B-domain deletions).

## Conclusions

For the first time in studies of FEIs in HA patients, we employed a statistical method that appropriately accounted for the non-independence of data due to both the sizeable genetic relatedness and *F8* mutational heterogeneity which exists when subjects with Mendelian loss-of-function disorders are sampled. By employing this method in PATH, on IC-genotypes of variations (i.e., mainly SNPs but some SNVs with rare MAs and/or racially-restricted MAs) assigned to pleiotropic-IMD-loci known to influence risk of developing two or more AADs, we identified: (i) the novel contribution of two genes (*NOS2A* and *B3GNT2*) to the risk of HA patients having FEIs on entering the study; (ii) additional genetic determinants of baseline-FEI-status in two pleiotropic-IMD-genes (*CTLA4* and *IL10* or, more accurately, the *IL10*-gene-superfamily) previously known to influence FEI risk (i.e., historical-FEI-status); and (iii) an interaction between race and a *B3GNT2*-assigned SNP that may contribute to the greater risk of FEI development long known to occur in HA patients with self-reported BA-racial-ancestry. Finally, we established that a relatively sizeable genetic component of baseline-FEI-status remains to be identified and is likely due to gene-based variants with rare MAs. As such, identification of these currently unknown rare genetic variants will require larger studies with more related HA patients and either whole-exome- or whole-genome-sequencing to identify (and genotype) the rare variants not included on GWAS-chips.

## Supplementary Material

Supplement 1

## ABBREVIATIONS

HA: Hemophilia A
F: factor
FVIII: factor VIII
F8: FVIII gene
tFVIIIs: therapeutic FVIII proteins
FEIs: FVIII-inhibitors
IMD: immune-mediated-disease
AADs: autoimmune-/autoinflammatory-disorders
FVIII:C: FVIII coagulant activity
pd: plasma-derived
r: recombinant
I: intron
invs: inversions
I22-invs: intron-22 inversions
SBSM: single-base-substitution-mutation
HTC: hemophilia-treatment center
PATH: Personalized Alternative Therapies for Hemophilia
BA: black-African
WE: white-European
SNPs: single-nucleotide-polymorphisms
SNVs: single-nucleotide-variants
IC: ImmunoChip
GCAS: gene-centric association-scan
ORs: odds ratios
ns: nonsynonymous
PTPs: previously-treated patients
Chr: chromosome
NO: nitric oxide
NOS: NO-synthase
MS: multiple sclerosis
IDDM: insulin-dependent diabetes mellitus
RA: rheumatoid-arthritis
SLE: systemic-lupus-erythematosus
MG: myasthenia gravis
MAs: minor alleles
GD: Graves’-disease
AS: ankylosing spondylitis
CD: Crohn’s disease
IL: interleukin
IL10: IL-10 gene
B3GNT2: UDP‐GlcNAc:bGal b-1,3-N-acetyl-glucosaminyltransferase-2
CTLA4: cytotoxic lymphocyte-associated-protein-4
CI: confidence interval
LB: lower bound
UB: upper bound
IT: immunologic tolerance
IR: immunologic reactivity
MA: minor allele
MAFs: MA frequencies
