# Supplementary material for: A Scan of Pleiotropic Immune Mediated Disease Genes Identifies Novel Determinants of Baseline FVIII Inhibitor Status in Hemophilia-A
Source: Res Sq. 2023 Oct 18:rs.3.rs-3371095. Preprint. [Version 1] doi: 10.21203/rs.3.rs-3371095/v1 (PMC10602130; doi:10.21203/rs.3.rs-3371095/v1)
Supplement: Supplement 1 [file NIHPPrs3371095v1-supplement-1.pdf]

Supplementary Files

This is a list of supplementary files associated with this preprint. Click to download.

- AlmeidaetalSupplementalAppendixGenesnImmunity09212023FinalwoIllustrations.docx
- SupplementaryFigure1PATH1GandIFinal.tif
- SupplementaryFigure2PATH1GandIFinal.tif
- Table1aAlmeidaetalPATH1GandIFinal09212023.tif
- Table1bAlmeidaetalPATH1GandIFinal09212023.tif
- Table2AlmeidaetalPATH1GandIFinal09212023.tif
- TABLES.docx
